# The association between perfectionism and academic procrastination among undergraduate nursing students: The role of self‐efficacy and resilience

**DOI:** 10.1002/nop2.1922

**Published:** 2023-07-18

**Authors:** Haitao Huang, Yueming Ding, Yipei Liang, Xiao Wan, Qianwen Peng, Yiming Zhang, Deren Kong, Chaoran Chen

**Affiliations:** ^1^ Institute of Nursing and Health, School of Nursing and Health Henan University Kaifeng China; ^2^ School of Business Henan University Kaifeng China

**Keywords:** academic procrastination, perfectionism, resilience, self‐efficacy, undergraduate nursing students

## Abstract

**Aims:**

This study aimed to investigate the associations between perfectionism and undergraduate nursing students' academic procrastination, the mediating effects of self‐efficacy and the moderating role of resilience.

**Design:**

A cross‐sectional survey.

**Methods:**

The survey was conducted from March to May 2022 with a sample of 587 undergraduate nursing students in two undergraduate universities in China. A descriptive statistical approach, Pearson's correlation analysis and the Hayes' PROCESS Macro model 4 and 14 were used to analyse the available data.

**Results:**

Adaptive perfectionism and maladaptive perfectionism negatively and positively predicted academic procrastination in nursing undergraduates, respectively. Self‐efficacy played a partially mediating role in the association between adaptive perfectionism and academic procrastination. Furthermore, resilience played a moderating role in the association between adaptive perfectionism and academic procrastination.

**Conclusions:**

Maladaptive perfectionism and low levels of resilience and self‐efficacy may increase the risk of academic procrastination among nursing undergraduates. Nursing educators can take measures to decrease the risk of academic procrastination among nursing undergraduate students by guiding them to cultivate adaptive perfectionism tendencies and improve their self‐efficacy and resilience.

**Impact:**

The findings of this study can be used to develop targeted coping and prevention measures for nursing educators to reduce the incidence of academic procrastination among nursing undergraduates.

**Patient or Public Contribution:**

Five hundred eighty‐seven undergraduate nursing students from two undergraduate universities participated in the study and responded to questions on perfectionism and academic procrastination, etc.

## 1 INTRODUCTION

Procrastination refers to nonadaptive behaviour in which people involuntarily postpone a predetermined plan without a clear reason (Johansson et al., 2023). Academic procrastination is a form of procrastination in school situations and is related to the fulfilment of studying tasks (Huang, Ding, Liang, et al., 2022). Some scholars posit that academic procrastination is a conscious decision to postpone tasks that should be completed within the designated timeframe although that such a delay may have deleterious effects (Gustavson & Miyake, 2017). Other academics construe academic procrastination as the emotional distress experienced by individuals who put off undertaking a task that must eventually be accomplished (Flett et al., 2012). Academic procrastination is common among medical college students, with approximately 13.8% to 49.9% of medical college students reporting procrastination on learning tasks (Madhan et al., 2012; Mortazavi et al., 2015). Studies have demonstrated that academic procrastination not only results in a decrease in academic performance and adversely affects college students' approach to learning but also engenders negative emotions, such as depression, anxiety, and shame, and can even be a contributing factor to suicide (Balkis & Duru, 2016; Flett et al., 2016; Huang, Ding, Zhang, et al., 2022; Martinčeková & Enright, 2020; Yang et al., 2022). Furthermore, academic procrastination can drain students and hinder them from gaining further nursing knowledge and expertise (Shaw et al., 2016), which is detrimental to the training of nurses and the quality of nursing education. Currently, the burgeoning demand for care and the escalating global difficulties in recruiting and retaining nurses are undermining nursing results worldwide (Donaghy et al., 2022; Yu et al., 2022). Thus, it is critical to train more professional and enthusiastic nursing students. Consequently, it is necessary for educators to investigate the risk factors and the mechanisms associated with academic procrastination among nursing students because this information may facilitate the formation of coping and prevention approaches that can enable the global training of more superior nursing students.

## 2 BACKGROUND

### 2.1 Perfectionism and academic procrastination

Perfectionism is a specific personality trait that is typically regarded as multidimensional, encompassing maladaptive perfectionism and adaptive perfectionism (Gärtner et al., 2020; Smith et al., 2019). The former involves a cognitive deficiency characterized by a tendency to be overly critical of one's own performance, whereas adaptive perfectionism refers to the achievement of high‐quality positive goals driven by positive internal reinforcement and a desire to accomplish tasks (Gärtner et al., 2020). Studies have confirmed that the proportion of perfectionists among university students is very high, and approximately 2/3 of students can be considered perfectionists (Grzegorek et al., 2004). Studies on the association between perfectionism and procrastination have found that certain components of perfectionism are significantly predictive of procrastination. In the development of the Frost Multidimensional Perfectionism Scale (FMPS), Frost et al. examined the relationship between perfectionism and procrastination and found that in the six subscales, concern over mistakes was significantly correlated with attitude towards procrastination, personal standards and organization were significantly correlated with procrastination frequency, and parental expectations and criticism were significantly correlated with both procrastination frequency and attitude (Frost et al., 1993). Abdollahi et al.'s (2020) findings also indicated that personal standards perfectionism and academic hardiness had negative relationships with academic procrastination, whereas evaluative concerns perfectionism had a positive relationship with academic procrastination. Studies with Chinese participants have found similar results (Chen et al., 2013; Chi et al., 2012).

Although some studies have documented a link between perfectionism and procrastination, these studies have rarely involved nursing undergraduates. In China, nursing undergraduates play an increasingly important role in nursing education (Fu et al., 2022). Data from China show that registered nurses with advanced diplomas or bachelor's degrees are the most needed workforce at all levels of health care (including tertiary and secondary hospitals) and in primary care departments (Fu et al., 2022; Wang et al., 2016). Undergraduate nursing students are likely to have higher expectations for the future than nursing students with junior college diplomas. The specialty of nursing and the future professional environment also present high requirements for undergraduate nursing students, so these students may have a high tendency towards perfectionism (Ma et al., 2014). Given these findings, we postulate the following hypothesis:

Hypothesis 1 Perfectionism can directly affect the academic procrastination of undergraduate nurses, adaptive perfectionism can negatively predict academic procrastination, and maladaptive perfectionism can positively predict academic procrastination.

### 2.2 The potential mediating effect of self‐efficacy

Self‐efficacy is an individual's belief, judgement, or self‐perception of his or her ability to successfully execute a particular behaviour at a certain level prior to performing the action (Whitehall et al., 2021). According to Bandura's theory of self‐efficacy, the level of self‐efficacy is mainly influenced by experiences of success or failure, demonstration effects, emotional states, and physiological arousal (Bandura et al., 1999). As a distinct personality trait, perfectionism has been shown to be closely related to self‐efficacy (Jaworski et al., 2022). Adaptive perfectionism can increase self‐efficacy levels, while maladaptive perfectionism can decrease self‐efficacy levels (Luo & Liu, 2019).

Self‐efficacy is also closely related to academic procrastination. Social cognitive theory suggests that self‐efficacy occupies a central position and has universal significance in the various mechanisms of behaviour (Young et al., 2014). Whether an individual can make the effort to achieve desired goals depends on his or her core beliefs about the ability to engage in the behaviour, while other factors serve only as references and motivation (Salles, 2017). A meta‐analysis of 156 studies showed that self‐efficacy is one of the important influencing factors of procrastination (Steel, 2007). Maddux's research showed that self‐efficacy affects people's upcoming actions and efforts to achieve goals (Maddux & Gosselin, 2012). Students with high self‐efficacy tend to view tasks, difficulties, and setbacks as opportunities for growth and are more likely to adopt a problem‐focused approach when dealing with academic pressure (Denovan & Macaskill, 2017). As a result, these students are more likely to experience success from their efforts, thus boosting their confidence and enabling them to make greater progress (Høigaard et al., 2015). In contrast, students with low self‐efficacy are less likely to engage in effective proactive learning behaviours (Guo et al., 2019). Combined with the above views, we propose the following hypothesis:

Hypothesis 2 Self‐efficacy mediates the association between perfectionism and academic procrastination among undergraduate nursing students.

### 2.3 The potential moderating effect of resilience

Resilience is defined as “an individual's behavioral tendency to adapt to changing circumstances and the ability to recover from stressful situations” (Bhatnagar, 2021). As an individual stress coping resource, resilience can help individuals effectively resist the negative effects of stress, so it has become an important issue in positive psychology (Troy et al., 2023). While perfectionism may play a role in academic procrastination, overall, it does not affect everyone equally. That is, individuals with different levels of resilience may have different levels of correlation strength between perfectionism, academic procrastination, and self‐efficacy. According to the ‘broaden‐and‐build theory’ (BBT) of positive emotions, individuals in a positive emotional state can think broadly, recognize events with a positive attitude, find the positive meaning of events (even adverse events), and have stronger motivation and ability to adapt to the environment (Fisher et al., 2022). This often produces positive results, expands the scope of people's attention, cognition and behaviour, enables people to obtain and analyse information more effectively and make more appropriate action choices and enhances psychological adaptability (Fredrickson & Joiner, 2018). Previous studies have shown that resilience is associated with perfectionism and can mitigate its effects (Çerkez, 2017; Chen et al., 2022). In addition, resilience is thought to be an important protective factor for positive psychology in humans and is associated with positive outcomes, such as reduced procrastination (Ko & Chang, 2019). Therefore, we believe that resilience is a plausible moderator in the relationship between perfectionism and academic procrastination. Based on the above arguments, we postulate the following hypothesis:

Hypothesis 3 Resilience moderates the association between perfectionism and academic procrastination among undergraduate nursing students.

## 3 THE STUDY

### 3.1 Aims

**FIGURE 1:**
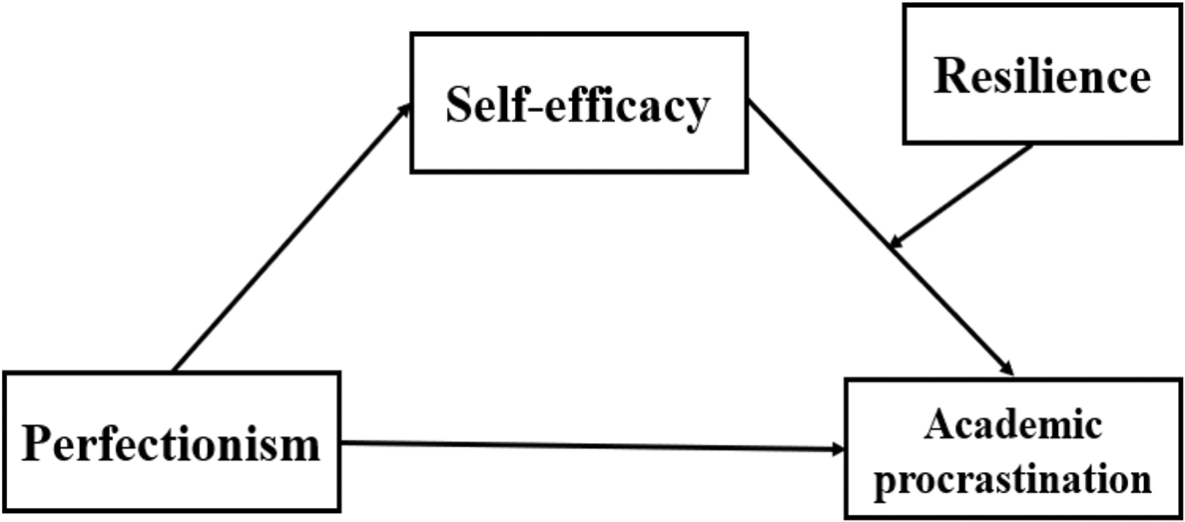
The theoretical model of this study.

In this study, we first investigate the association between perfectionism and academic procrastination among undergraduate nursing students. Then, we investigate the mediating role of self‐efficacy in the association between perfectionism and academic procrastination. Finally, we investigate the main effect of resilience with a particular focus on its moderating role in the relationship between perfectionism and academic procrastination. The proposed theoretical model to be tested is shown in Figure 1.

### 3.2 Design

A cross‐sectional design was used for this research.

### 3.3 Participants

The participants were recruited from two undergraduate universities in Henan, PR China, from March to May 2022. Nursing undergraduates who satisfied the following inclusion criteria were recruited by convenience sampling: (1) full‐time nursing undergraduates in grade 1, grade 2 and grade 3 and (2) understood the purpose of this study and expressed intention to participate. The exclusion criteria on was failure to complete all questionnaires for various reasons. All undergraduate nursing students who met the inclusion criteria were given questionnaires. Finally, a total of 587 questionnaires were analysed after excluding four incomplete questionnaires.

### 3.4 Data collection

Before sampling, we discussed the contents and procedures of the questionnaire with the psychological services departments at each university. Investigators began handing out paper questionnaires to students as they gathered in a classroom (approximately 50 students at a time). Participants were not given any incentive or inducement throughout the test. Participants were told that their answers to the questionnaire would be anonymous and confidential and that the data collected would only be used for academic research.

### 3.5 Ethical considerations

This study was reviewed and approved by the Institutional Review Board of Henan Provincial Key Laboratory of Psychology and Behavior (reference: 20220107001) and performed in accordance with the Declaration of Helsinki. All participants gave voluntary written informed consent prior to study participation. All methods were performed in accordance with the relevant guidelines and regulations.

### 3.6 Instruments

#### 3.6.1 Demographic information

A demographic questionnaire assessed participant characteristics including age, gender, and home location.

#### 3.6.2 The Chinese frost multidimensional perfectionism scale (CFMPS)

The Frost Multidimensional Perfectionism Scale (FMPS), compiled by Frost et al. (1990) was designed to measure the typical cognitive, emotional, and behavioural expressions of perfectionists. Zi and Zhou (2006) further revised the FMPS in 2006 to accommodate the culture and language of mainland China, resulting in the Chinese version of the FMPS. The Chinese version of the FMPS consists of 27 items divided into five dimensions: Concern over Mistakes (6 items, e.g. ‘If I fail in my studies, it means I am a failure’), Organization (6 items, e.g. ‘It is important to me to be organized in my work’), Parental Expectations (5 items, e.g. ‘My parents set high standards for me’), Personal Standards (6 items, e.g. ‘I might become a second‐class person if I do not set the highest standards for myself’), and Doubts about Actions (4 items, e.g. ‘I often hesitate over even small everyday matters’). ‘Organization’ was considered adaptive perfectionism. ‘Concern over Mistakes’, ‘Parental Expectation’, ‘Personal Standards’ and ‘Doubts about Actions’ were considered maladaptive perfectionism. Five Likert scales were used for the scale. The scale has been proven to have good reliability and validity (Zi & Zhou, 2006). In this study, the overall internal consistency reliability of the questionnaire was 0.83, and the internal consistency reliabilities of adaptive and maladaptive perfectionism were 0.81 and 0.85, respectively.

#### 3.6.3 Aitken procrastination inventory (API)

The API is a self‐assessment scale developed by Aitken in 1982 to evaluate the long‐term persistent academic procrastination behaviour of college students (Aitken, 1982). It has a total of 19 items and has been proven to have good reliability and validity in the Chinese context (Chen et al., 2008). The API is scored on a 5‐point Likert scale, with ‘1’ meaning ‘completely inconsistent’ and ‘4’ meaning ‘completely consistent’. Items 2, 4, 7, 11, 12, 14, 16, 17 and 18 are inversely scored. Sample items are ‘I always start things at the last minute; I'm very careful about returning library books on time’. A high score indicates a higher degree of academic procrastination. In this study, the Cronbach's *α* of the API was 0.82.

#### 3.6.4 General self‐efficacy scale (GSEC)

The GSEC was chosen to measure self‐efficacy (Schwarzer et al., 1999). The scale is a one‐dimensional scale with 10 items, such as ‘If I try my best, I can always solve the problem’. A 4‐point Likert scale was used for scoring, where 1 ~ 4 points indicated ‘completely inconsistent’ to ‘completely consistent’, respectively. The average score of all items was calculated; the higher the score was, the higher the self‐efficacy. The Chinese version of the GSEC has been proven to have good reliability with a Cronbach's coefficient was 0.89 (Li et al., 2019). In this study, the Cronbach's *α* of the GSEC was 0.89.

#### 3.6.5 Connor‐Davidson resilience scale (CD‐RISC)

The CD‐RISC was developed by Connor and Davidson in 2003. It includes 25 items and is divided into five dimensions: tenacity, tolerance of negative effects, positive acceptance of change, control and spiritual influences (Connor & Davidson, 2003). The Chinese version of the CD‐RISC was revised by Yu (Yu & Zhang, 2007), and 25 items of the original scale were retained and adjusted it to three dimensions: tenacity, strength and optimization. Five‐point Likert scales were used, and each item was assigned according to the degree of conformity with the participants' own situation (0 = never, 4 = almost always). The CD‐RISC (Chinese version) was tested in the general population in China (Cronbach's *α* = 0.91) (Yu & Zhang, 2007). The Cronbach's *α* of the CD‐RISC in the current study was 0.91.

### 3.7 Data analysis

All data were analysed using IBM SPSS statistics 25.0 and the PROCESS macro3.3. PROCESS provides ordinary least‐squares, regression‐based path analysis such as structural equation modelling but supplies additional useful statistics and safeguards against irregular sampling distributions (Hayes, 2017). The demographic characteristics of the participants were represented by descriptive statistics. Pearson correlation analysis was used to investigate the relationship between perfectionism, self‐efficacy, resilience, and academic procrastination. Harman's single‐factor test was applied to evaluate the common method bias derived from self‐reported data (Podsakoff et al., 2003). The mediating role of self‐efficacy was examined using PROCESS Model 4. The moderating effect of resilience was analysed using PROCESS Model 14 (Hayes, 2017). In addition, we used the 5000 resample bootstrapping method with a 95% CI to test the effect of the independent variables on the dependent variable through the mediating variable. Gender, age, home location, grade, and family structure were controlled as covariate variables in the model. It is assumed that the 95% deviation corrected confidence interval (CI) did not contain zero, indicating that the effect was statistically significant.

### 3.8 Validity and reliability/rigour

All the measuring tools used for testing were adjusted and verified for Chinese culture and had good validity and reliability. In this research, the Cronbach's *α* values of the CFMPS, API, GSEC, and CD‐RISC were 0.83, 0.82, 0.89, and 0.91, respectively. In addition, before the formal investigation, all investigators were trained on registration, checking the completeness of questionnaires, and ethical tenets of conducting research. To reduce the risk of self‐reported bias, the identities of all participating nursing undergraduates were kept strictly confidential. Finally, to ensure the rigour and accuracy of the statistical analysis, we invited a statistics professor to examine the data processing.

## 4 RESULTS

### 4.1 Common method bias tests

Harman's single‐factor test extracted 19 factors with eigenvalues >1. The first factor explained 17.06% of the total variance, which was below the recommended threshold of 40% (Podsakoff et al., 2003). This suggests that common method bias was unlikely to confuse the interpretation of the data analysis results.

### 4.2 Characteristics of the participants

As shown in Table 1, a total of 587 undergraduate nursing students effectively participated in the survey, including 141 males (24%) and 446 females (76%). The participants' ages ranged from 17 to 24 years old (mean = 19.65, SD = 1.11). A total of 37.1% of the undergraduate nursing students came from towns, while the remaining 62.9% came from the villages. A total of 49.7% of the nursing students were freshmen, 39.7% were sophomores, and 10.6% were juniors. Finally, 14.8% of the participants were only children, and the vast majority (85.2%) of participants came from families with multiple children.

**TABLE 1:** Demographic characteristics of undergraduate nursing students (*N* = 587).

Variable	*N* = 587	%
Gender, *n* (%)		
Male	141	24
Female	446	76
Age, M (SD)	19.65 (1.11)	
Grade		
Grade 1	292	49.7
Grade 2	233	39.7
Grade 3	62	10.6
Home location		
Town	218	37.1
Village	369	62.9
Only child in family		
Yes	87	14.8
No	500	85.2

### 4.3 Descriptive analysis and correlations between overall variables

Then means, standard deviations (SD), and Pearson correlations of each study variable are shown in Table 2. The results show that adaptive perfectionism was significantly positively correlated with self‐efficacy (*r* = 0.250, *p* < 0.01) and resilience (*r* = 0.353, *p* < 0.01, higher CFMPS score indicating a high level of resilience), while it was significantly negatively correlated with academic procrastination (*r* = −0.475, *p* < 0.01). Moreover, maladaptive perfectionism was significantly negatively correlated with self‐efficacy (*r* = −0.181, *p* < 0.01) and resilience (*r* = −0.325, *p* < 0.01), while self‐efficacy was significantly positively correlated with resilience (*r* = 0.665, *p* < 0.01). Finally, maladaptive perfectionism was significantly positively correlated with academic procrastination (*r* = 0.132, *p* < 0.01).

**TABLE 2:** Means, standard deviations, and correlations of the study variables (*N* = 587).

Variables	Mean ± SD	1	2	3	4	5	6	7	8	9	10	11	12	13
Perfectionism	3.16 ± 0.47	1												
CM	2.38 ± 0.86	0.703**	1											
PE	3.21 ± 0.72	0.604**	0.302**	1										
PS	3.04 ± 0.72	0.787**	0.450**	0.329**	1									
DA	3.26 ± 0.75	0.617**	0.446**	0.223**	0.326**	1								
OR	3.93 ± 0.68	0.421**	−0.133**	0.114**	0.302**	0.089*	1							
MP	2.98 ± 0.55	0.942**	0.796**	0.634**	0.723**	0.694**	0.111**	1						
SE	2.52 ± 0.53	0.164**	−0.044	0.124**	0.301**	−0.150**	0.250**	−0.181**	1					
Resilience	3.45 ± 0.50	0.100*	−0.185**	0.093*	0.224**	−0.171**	0.353**	−0.325**	0.665**	1				
Tenacity	3.38 ± 0.56	0.092*	−0.161**	0.104*	0.216**	−0.185**	0.308**	−0.019	0.642**	0.950**	1			
Strength	3.60 ± 0.54	0.097*	−0.193**	0.06	0.216**	−0.129**	0.360**	−0.029	0.571**	0.904**	0.762**	1		
Optimization	3.38 ± 0.57	0.069	−0.133**	0.063	0.129**	−0.100*	0.265**	−0.024	0.510**	0.729**	0.567**	0.623**	1	
AP	2.56 ± 0.51	−0.044	0.241**	0.065	−0.142**	0.175**	−0.475**	0.132**	−0.181**	−0.325**	−0.295**	−0.354**	−0.168**	1

Abbreviations: AP, Academic Procrastination; CM, Concern over Mistakes; DA, Doubts about Actions; MP, Maladaptive Perfectionism; OR, Organization; PE, Parental Expectations; PS, Personal Standards; SE, Self‐Efficacy.

* *p* < 0.05

** *p* < 0.01.

### 4.4 Testing the mediating effect of self‐efficacy

PROCESS macro Model 4 was used to analyse the mediating role of self‐efficacy. Adaptive perfectionism significantly negatively predicted academic procrastination after controlling for gender, age, home location, grade, and family structure (c = −0.476, *t* = −12.928, *p* < 0.001). When adaptive perfectionism and self‐efficacy were used in the regression equation together, the predictive effect of adaptive perfectionism on academic procrastination was still significant (c’ = −0.454, *t* = −11.956, *p* < 0.001). Adaptive perfectionism had a significant positive predictive effect on self‐efficacy (a = 0.256, *t* = 6.497, *p* < 0.001), and self‐efficacy had a significant negative predictive effect on academic procrastination (b = −0.084, *t* = −2.169, *p* < 0.05). This manifested in self‐efficacy partially mediating the relationship between adaptive perfectionism and academic procrastination. The bootstrap method test with percentile bias correction indicated that self‐efficacy had a significant mediating effect between adaptive perfectionism and academic procrastination, with ab = −0.022, boot SE = 0.011, and 95% CI = (−0.045, −0.002).

**FIGURE 2:**
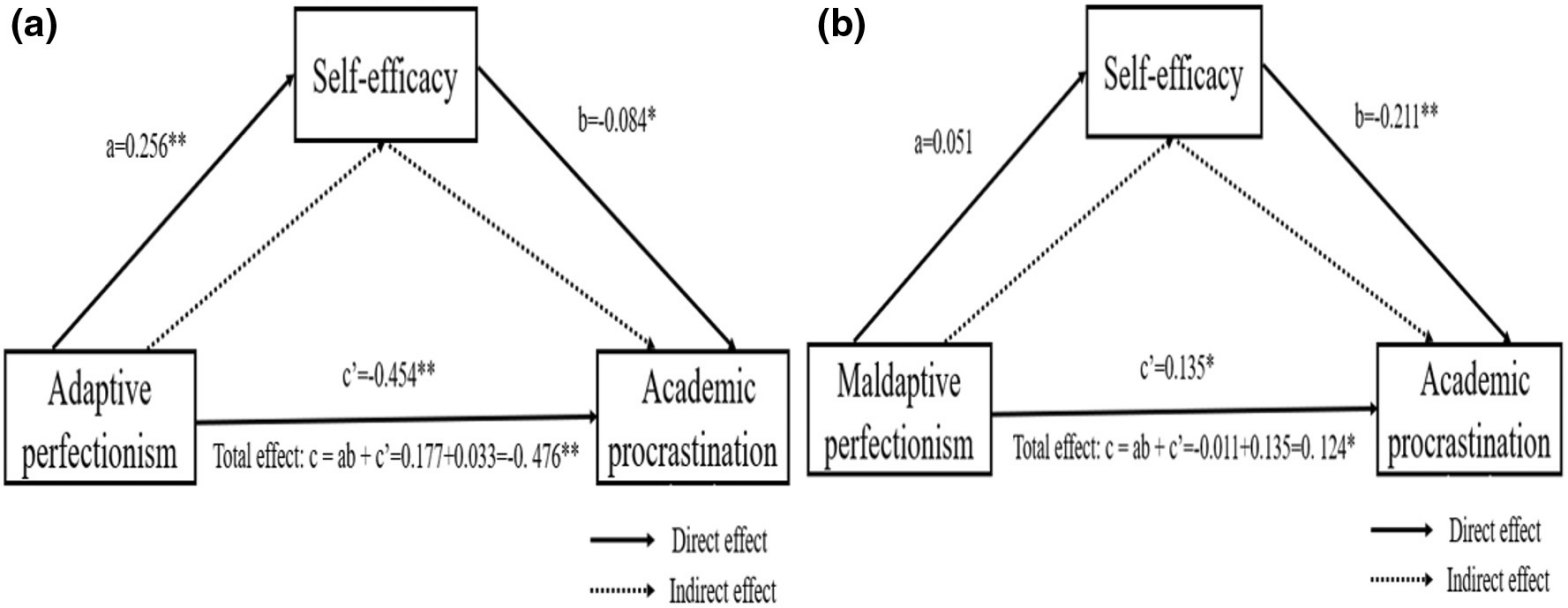
The mediating effect of self‐efficacy (a): the mediating effect of self‐efficacy between adaptive perfectionism and academic procrastination; (b): the mediating effect of self‐efficacy between maladaptive perfectionism and academic procrastination. *p < 0.05; **p < 0.01.

Maladaptive perfectionism significantly positively predicted academic procrastination after controlling for gender, age, home location, grade, and family structure (c = 0.124, *t* = 3.004, *p* < 0.05). When maladaptive perfectionism and self‐efficacy were used in the regression equation together, the predictive effect of maladaptive perfectionism on academic procrastination was still significant (c’ = 0.135, *t* = 3.332, *p* < 0.05). Self‐efficacy had a significant negative predictive effect on academic procrastination (b = −0.211, *t* = −5.117, *p* < 0.001). However, maladaptive perfectionism had no statistically significant effect on self‐efficacy (a = 0.051, *t* = 1.256, *p* > 0.05). This suggests that maladaptive perfectionism was only directly associated with academic procrastination. Therefore, Hypothesis 1 was supported. Hypothesis 2 was partially supported; that is, self‐efficacy only mediated the relationship between adaptive perfectionism and academic procrastination as a mediating variable. Figure 2 shows the direct, indirect, and total effects.

### 4.5 Moderating effect analysis

PROCESS macro Model 14 was used to analyse the moderating role of resilience in the relationship between adaptive perfectionism and academic procrastination. As shown in Table 3, the interaction terms between self‐efficacy and resilience had significant predictive influence on the academic procrastination of undergraduate nursing students (*β* = −0.105, *p* < 0.05). This suggests that resilience moderates the association between adaptive perfectionism and academic procrastination.

**TABLE 3:** Moderating role of resilience in the relationship between adaptive perfectionism and academic procrastination.

Predictive variable	Model 1 (criterion: Academic procrastination)		Model 2 (criterion: Self‐efficacy)		Model 3 (criterion: Academic procrastination)	
	*β*	*t*	*β*	*t*	*β*	*t*
Adaptive perfectionism	−0.476	−12.928**	0.256	Self‐efficacy**	−0.454	−11.956**
Self‐efficacy					−0.084	−2.169*
Resilience					−0.211	−4.202**
Self‐efficacy × Resilience					−0.105	−3.018*
*R* ^2^	0.230		0.115		0.274	
*F*	28.886**		12.512**		24.245**	

* *p* < 0.05

** *p* < 0.01.

**FIGURE 3:**
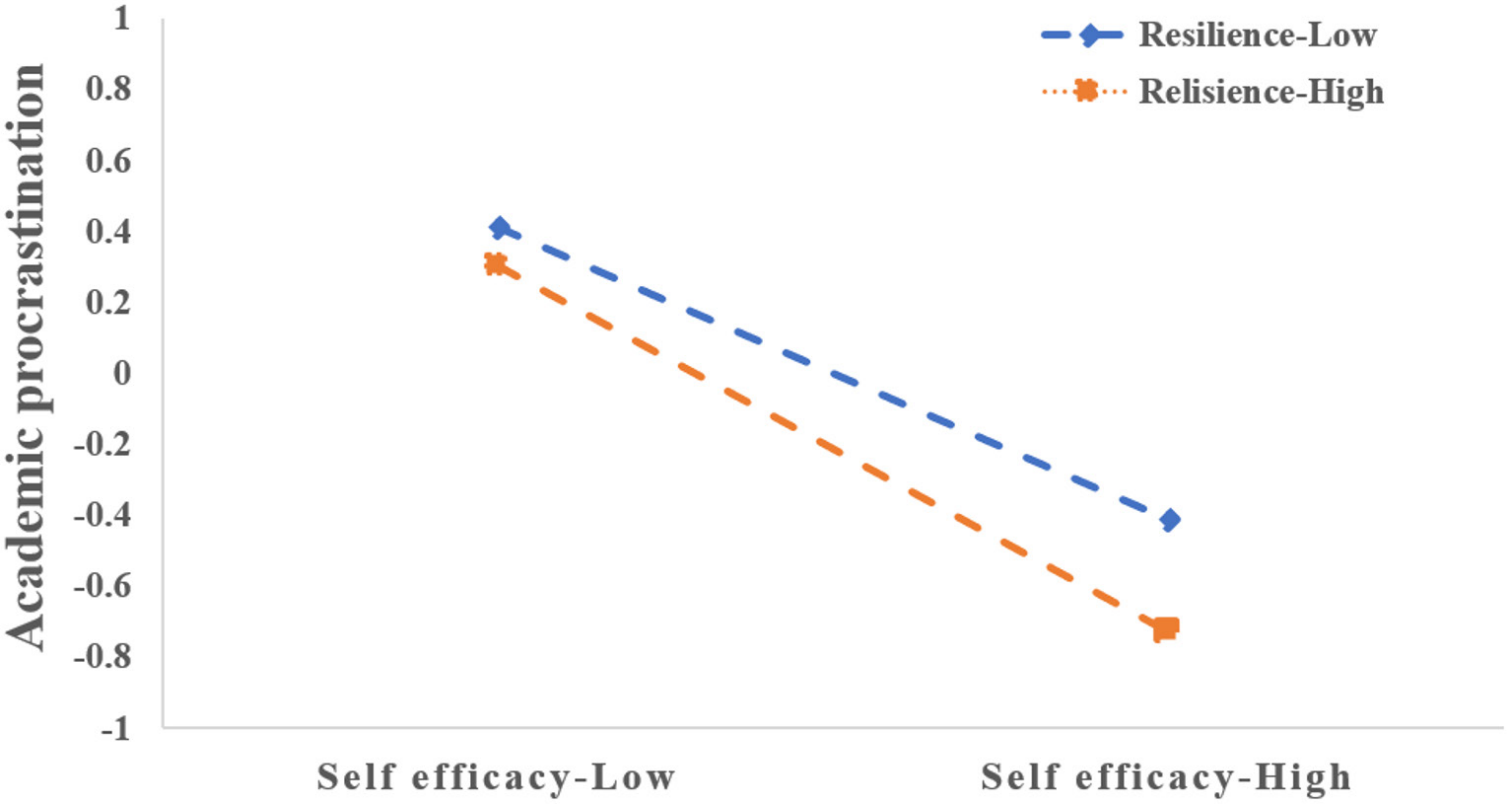
The moderating role of resilience between adaptive perfectionism and academic procrastination.

Through simple slope analysis, the adjustment effect of different levels of resilience can be shown more intuitively. Whether resilience was at a high or low level, adaptive perfectionism had a negative effect on academic procrastination. The difference was that for undergraduate nursing students with high resilience, the effect of adaptive perfectionism on academic procrastination showed an obvious trend of strengthening (*β* = −0.517, *t* = −9.863, *p* < 0.001). However, for undergraduate nursing students with low resilience, the effect of adaptive perfectionism on academic procrastination was still significant but decreased (*β* = −0.306, *t* = 5.962, *p* < 0.001). Figure 3 shows the moderating role of resilience between adaptive perfectionism and academic procrastination.

## 5 DISCUSSION

In the current study, we investigated the relationship between perfectionism and academic procrastination and its underlying mechanism among Chinese undergraduate nursing students. The results showed that there was a direct correlation between adaptive perfectionism and academic procrastination as well as between maladaptive perfectionism and academic procrastination. Self‐efficacy was the mediating factor between adaptive perfectionism and academic procrastination, and there was a partial mediating relationship. Resilience played a moderating role in the relationship between adaptive perfectionism and academic procrastination. The findings can help us further understand the relationship between perfectionism and academic procrastination and help relevant institutions take measures to further alleviate and improve the academic procrastination phenomenon among nursing undergraduates.

### 5.1 The relationship between perfectionism and academic procrastination

In support of Hypothesis 1, our study found that maladaptive perfectionism and adaptive perfectionism significantly positively and negatively predicted academic procrastination, respectively, which is consistent with previous research results (Abdollahi et al., 2020; Kurtovic et al., 2019). Numerous studies have shown that individuals often procrastinate on tasks that they should complete even though they are aware of the negative consequences, mainly because they fear harsh judgement (Burka & Yuen, 2007). These individuals tend to be governed by two irrational beliefs: on the one hand, they feel that their abilities are insufficient to accomplish tasks; on the other hand, they perceive the world as too difficult and demanding. Fear of failure, negative perfectionism, and evaluation anxiety are manifestations of these irrational beliefs (Steel, 2007; Steinert et al., 2021).

Maladaptive perfectionists have high or unrealistic standards for themselves. They view everything as either perfect or failing. They hesitate to act because they are terrified of failing and errors (Smith et al., 2019). These characteristics are consistent with the abovementioned irrational beliefs that govern procrastination. People who are troubled and harmed by these irrational beliefs are more likely to make negative or even catastrophic interpretations of benign events, thereby irrationally postponing many actions in their lives (Steel, 2007). In contrast, adaptive perfectionists seek reasonable and realistic success criteria and derive satisfaction and self‐affirmation from them (Suh et al., 2019). Some studies have suggested that procrastination is essentially a failure of self‐regulation or self‐control (Guo et al., 2019). Well‐organized adaptive perfectionists can reduce the frequency of procrastination by strengthening goal setting, narrowing the gap between intention and action, and improving their bad habits of procrastination (Steinert et al., 2021).

The particularity of nursing requires nursing undergraduates to have a rigorous working attitude to coordinate and deal with daily tasks. They need to check and recheck each nursing practice in school to avoid and reduce errors as much as possible; otherwise, they may bring serious consequences to patients and affect the quality of nursing (Cheng & Liu, 2017). Therefore, undergraduate nursing students gradually develop the tendency of perfectionism that needs to be repeatedly confirmed in the daily learning process. Maladaptive perfectionism may lead nursing students to be overly critical of perfection, resulting in greater stress. In this situation, they often choose to escape to alleviate their anxiety, thereby exhibiting more procrastination behaviour (Cheng & Liu, 2017). Nursing educators can help undergraduate nursing students establish correct cognition, recognize the significance, purpose and content of tasks, reduce their fear of failure, realize that failure is a normal phenomenon in life, summarize experience in a timely manner, and strive to do better next time. In addition, nursing educators should focus on cultivating nursing students' learning organization to reduce their procrastination behaviour.

### 5.2 The mediating role of self‐efficacy

Self‐efficacy was found to partially mediate the relationship between adaptive perfectionism and academic procrastination but not the relationship between maladaptive perfectionism and academic procrastination, which partially supports Hypothesis 2. This finding underlined adaptive perfectionism as an important factor for self‐efficacy and supported by previous literature (Razmi et al., 2020). Nursing undergraduates with adaptive perfectionism tend to be organized and plan, pursue success, and act decisively. They may often attain a high level of self‐efficacy and a positive self‐evaluation because they have accomplished challenging tasks (Steel, 2007). Driven by this internal motivation, they often experience joy in the learning process, which reduces the likelihood of procrastination. Conversely, when they have a high level of maladaptive perfectionism, nursing students fear failure and tend to have negative cognitions about the task process, which are likely to have a direct impact on academic procrastination.

### 5.3 The moderating role of resilience

Resilience was found to moderate the relationship between adaptive perfectionism and academic procrastination in nursing students but not the relationship between maladaptive perfectionism and academic procrastination, which partially supports Hypothesis 3. Specifically, high levels of resilience enhanced the effect of adaptive perfectionism on academic procrastination, which is similar to the conclusion of Hicks (Hicks & Wu, 2015). The reason may be related to the fact that individuals with different levels of resilience may adopt different coping strategies when facing problems (Okechukwu et al., 2022). For example, students with high levels of resilience prefer positive coping styles, which may change their perception of tasks and reduce perceived stress (Okechukwu et al., 2022) and, in turn, the frequency of academic procrastination. In addition, Villa pointed out that the identification and testing of moderator variables is affected by many factors, including sample size, sampling range, measurement error of the predictor variables that make up the interaction term, and the precision of the scale, which may reduce statistical power (Villa et al., 2003). This may be the reason why the moderating effect of resilience on the relationship between nonadaptive perfectionism and academic procrastination is not significant. Cleary et al. (2018) suggested that nursing students are in an important stage of development, and resilience is a necessary trait for them to achieve success in learning and practice. Therefore, enhancing the resilience of nursing students can not only strengthen the predictive effect of adaptive perfectionism on academic procrastination but also effectively reduce their risk of academic procrastination.

### 5.4 Implications for practice

The current research has important theoretical significance and practical value for the improvement and intervention of academic procrastination in nursing undergraduates. To reduce the academic procrastination of nursing students, the following suggestions are proposed. For education authorities, the prevention and reduction of academic procrastination should be part of a comprehensive education method, and the occurrence of academic procrastination in nursing students should be reduced by making scientific and reasonable education plans. For nursing educators, the first step is to guide undergraduate nursing students to form a positive perfectionism tendency by developing relevant courses or designing simple activities, such as individual counselling or group counselling. At the same time, educators should guide nursing students to carry out objective self‐awareness and self‐evaluation, enhance their sense of self‐worth, and improve their self‐awareness and respect. Finally, educators should cultivate nursing students' self‐efficacy and resilience to stimulate them to pursue reasonable and realistic standards, obtain satisfaction and self‐affirmation from these standards, and gradually eliminate the problem of academic procrastination.

### 5.5 Limitations

The results of this study are of great significance for the implementation of interventions aimed at reducing academic procrastination in nursing students. Although there are some highlights, several limitations must be considered. First, this study was a cross‐sectional study, so further longitudinal studies are needed to investigate the causal relationship between variables. Second, the data used in this study were all self‐reported by the subjects, which may affect the results of the study due to recall bias. Although deviation due to common methods was not found in this study, we can use a variety of data collection methods (such as a combination of self‐report and others' report) in future studies to ensure the reliability of research conclusions. Finally, the research objects of this study were only from two undergraduate universities, which hinders the generalizability of the research conclusion to some extent. Future studies can expand the sample sources and explore the differences in research results with different cultural backgrounds and educational levels.

## 6 CONCLUSIONS

Given the shortage of nurses worldwide and the increasing demand for nursing services, reducing the occurrence of academic procrastination among nursing students is crucial to reduce nursing loss and improve nursing quality. The results of this study showed that adaptive perfectionism and maladaptive perfectionism negatively and positively predicted academic procrastination in nursing undergraduates, respectively. Self‐efficacy played a partially mediating role in the association between adaptive perfectionism and academic procrastination. Moreover, resilience moderated the association between adaptive perfectionism and academic procrastination. Therefore, nursing educators can reduce the possibility of academic procrastination in nursing undergraduates by cultivating their adaptive perfectionism and improving their self‐efficacy and resilience.

## AUTHOR CONTRIBUTIONS

All authors made substantial contributions to conception and design, or acquisition of data, or analysis and interpretation of data. All authors were involved in drafting the manuscript or revising it critically for important intellectual content and approved the final version. Each author has participated sufficiently in the work to take public responsibility for appropriate portions of the content and agreed to be accountable for all aspects of the work in ensuring that questions related to the accuracy or integrity of any part of the work are appropriately investigated and resolved.

## FUNDING INFORMATION

This research was sponsored by Graduate Education Reform and Quality Improvement Project of Henan Province (Grant Number: YJS2021AL074), Graduate Education Innovation and Quality Improvement Project of Henan University (Grant Number: SYL19060141), and Philosophy and Social Science Planning Research Project of Kaifeng City (Grant Number: ZXSKGH‐2022‐1363).

## CONFLICT OF INTEREST STATEMENT

The authors declare that there are no conflicts of interest.

## ETHICS STATEMENT

This study has been reviewed and approved by Institutional Review Board of Henan Provincial Key Laboratory of Psychology and Behaviour (reference: 20220107001) and performed in accordance with the Declaration of Helsinki. All participants gave their voluntary written informed consent prior to study participation. All methods were performed in accordance with the relevant guidelines and regulations.

## Data Availability

The data presented in this study are available on request from the corresponding author.
